# Supplementary material for: Corticosterone Administration Alters White Matter Tract Structure and Reduces Gliosis in the Sub-Acute Phase of Experimental Stroke
Source: Int J Mol Sci. 2021 Jun 22;22(13):6693. doi: 10.3390/ijms22136693 (PMC8269094; doi:10.3390/ijms22136693)
Supplement: Supplementary file 1 [file ijms-22-06693-s001.zip › ijms-1266650-supplementary.pdf]

SUPPLEMENTARY MATERIAL

Corticosterone administration alters white matter tract structure and reduces gliosis in the sub-acute phase of experimental stroke

Katarzyna Zalewska, Rebecca J Hood, Giovanni Pietrogrande, Sonia Sanchez-Bezanilla, Lin Kooi Ong, Sarah J Johnson, Kaylene M Young, Michael Nilsson & Frederick R Walker

Supplementary figures

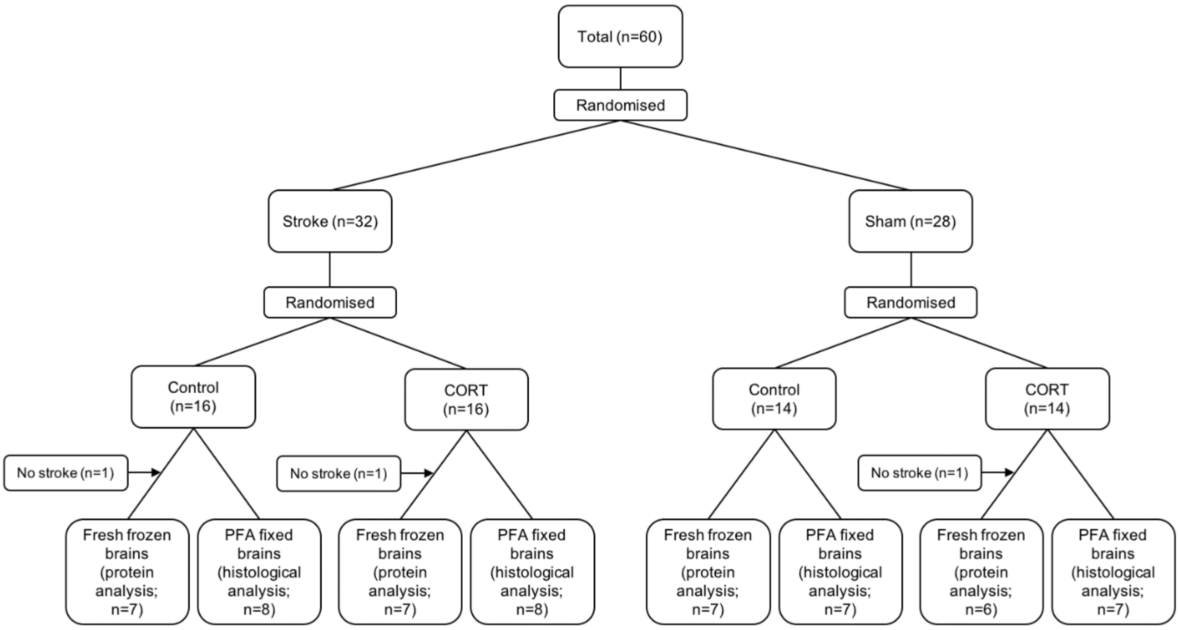

Supplementary Figure S1. Experimental Design – Inclusions/Exclusions. Diagram for inclusion and exclusion of mice in this study.

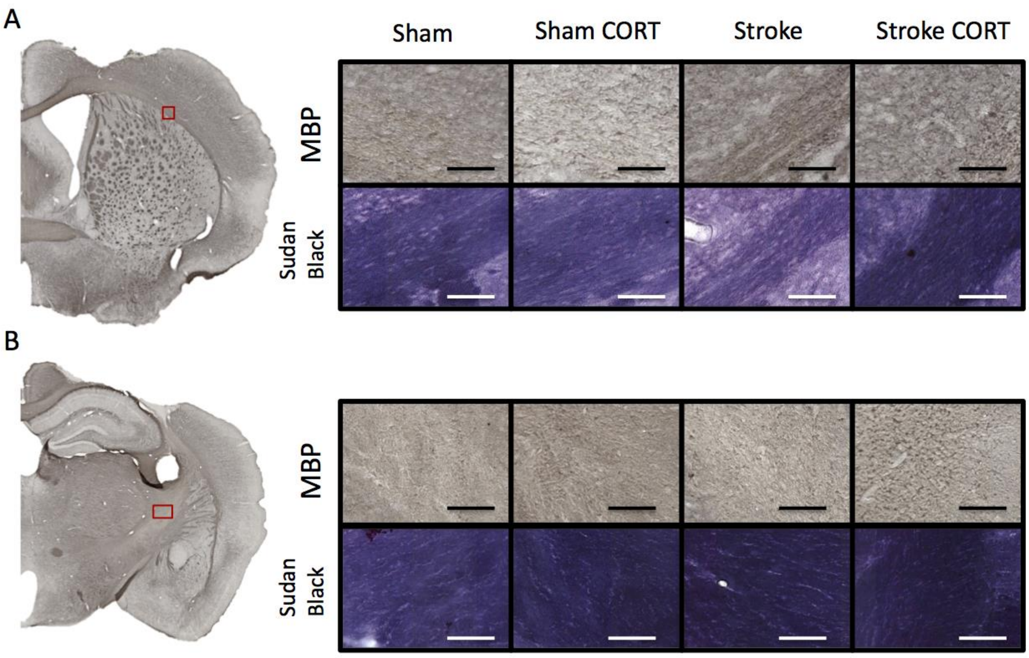

Supplementary Figure S2. Changes in myelin structure after stroke and CORT administration. (**A**) represents staining for MBP in CC with location of cropped region. Right Panel shows staining of MBP and Sudan Black from cropped region throughout different groups. Scale Bar = 75 µm for MBP and 100 µm for Sudan Black. (**B**) shows representative staining for MBP in CST region. Right Panel shows staining of MBP and Sudan Black from cropped region throughout different groups. Scale Bar = 100 µm for MBP and 150 µm for Sudan Black.

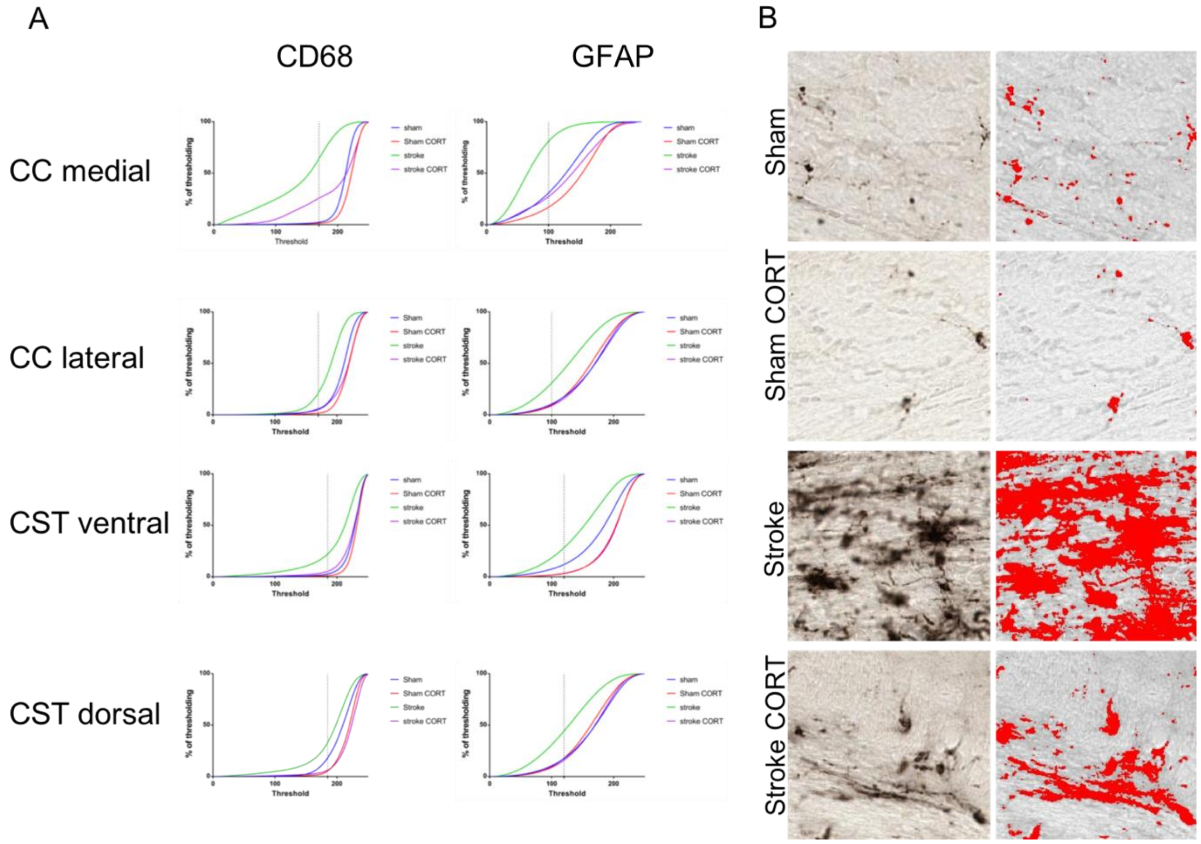

Supplementary Figure S3. The cumulative threshold spectra graphs. (**A**) shows cumulative threshold graphs for 2 regions of CC and 2 regions of CST for CD68 and GFAP. Dotted line represents pixel intensity which was used for further analyses. (**B**) shows representative CD68 staining from the CC region A. Right panel shows thresholding at PI = 170.
